# Proof of Concept Study: Comparability of Microbiome Diversity in Self- and Physician-Collected HPV-Positive and HPV-Negative Cervicovaginal Samples

**DOI:** 10.3390/ijms25115736

**Published:** 2024-05-24

**Authors:** Laura Asensio-Puig, Álvaro de Andrés-Pablo, Olfat Khannous-Lleiffe, Raquel Ibáñez, Amelia Acera, Silvia de Sanjosé, Toni Gabaldón, Laia Alemany, Laia Bruni, Miquel Àngel Pavón

**Affiliations:** 1 Cancer Epidemiology Research Programme, Catalan Institute of Oncology—Bellvitge Biomedical Research Institute (IDIBELL), 08908 L’Hospitalet de Llobregat, Spain; lasensio@idibell.cat (L.A.-P.); adeandres@idibell.cat (Á.d.A.-P.); raquelip@iconcologia.net (R.I.); lalemany@iconcologia.net (L.A.); lbruni@iconcologia.net (L.B.); 2 Programa de Doctorat en Biomedicina, Universitat de Barcelona (UB), 08036 Barcelona, Spain; 3 Barcelona Supercomputing Centre (BSC-CNS), 08034 Barcelona, Spain; olfat.khannous@bsc.es (O.K.-L.); toni.gabaldon@bsc.es (T.G.); 4 Institute for Research in Biomedicine (IRB), 08028 Barcelona, Spain; 5 Centro de Investigación Biomédica en Red de Epidemiología y Salud Pública (CIBERESP CB06/02/0073), 28029 Madrid, Spain; desanjose.silvia@gmail.com; 6 Atenció a la Salut Sexual i Reproductiva (ASSIR), SAP Cerdanyola-Ripollet, Institut Català de la Salut, 08291 Ripollet, Spain; amelia.acera@gmail.com; 7 ISGlobal, 08036 Barcelona, Spain; 8 Catalan Institution for Research and Advanced Studies (ICREA), 08010 Barcelona, Spain; 9 CIBER de Enfermedades Infecciosas, Instituto de Salud Carlos III, 28029 Madrid, Spain

**Keywords:** microbiome, HPV screening, self-sampling, nanopore sequencing, 16S rRNA

## Abstract

Recent studies have revealed the impact of human papillomavirus (HPV) infections on the cervicovaginal microbiome; however, few have explored the utility of self-collected specimens (SCS) for microbiome detection, obtained using standardised methods for HPV testing. Here, we present a proof-of-concept analysis utilising Oxford Nanopore sequencing of the 16S rRNA gene in paired samples collected either by the patient using an Evalyn Brush or collected by a physician using liquid-based cytology (LBC). We found no significant differences in the α-diversity estimates between the SCS and LBC samples. Similarly, when analysing β-diversity, we observed a close grouping of paired samples, indicating that both collection methods detected the same microbiome features. The identification of genera and *Lactobacillus* species in each sample allowed for their classification into community state types (CSTs). Notably, paired samples had the same CST, while HPV-positive and -negative samples belonged to distinct CSTs. As previously described in other studies, HPV-positive samples exhibited heightened bacterial diversity, reduced *Lactobacillus* abundance, and an increase in genera like *Sneathia* or *Dialister*. Altogether, this study showed comparable results between the SCS and LBC samples, underscoring the potential of self-sampling for analysing the microbiome composition in cervicovaginal samples initially collected for HPV testing in the context of cervical cancer screening.

## 1. Introduction

High-risk human papillomavirus (HR-HPV) infection, the most common sexually transmitted disease, is the primary cause of the development of cervical cancer [1]. It is estimated that over 80% of sexually active women during their lifetime will contract this infection, and while most infections will resolve spontaneously, a minority will progress to cervical dysplasia or cancer. In recent years, the incidence of cervical cancer in high-income countries has declined [2], largely due to the implementation of organised screening programs and human papillomavirus (HPV) vaccination. Strong evidence indicates that clinically validated HPV testing for oncogenic types is more accurate and cost-effective as a primary cervical cancer screening method than traditional cervical cytology (Pap smears) [3,4,5]. In addition, the introduction of self-sampling collection for HPV testing has emerged as a safe, accessible approach, increasing participation among women who might otherwise avoid sample collection by clinicians, therefore enhancing access to the screening test [6,7,8].

The community of microorganisms in a specific environment, also called microbiota, exerts an influence on the metabolic and immune environment of host tissues. Numerous factors such as ethnicity, genetics, lifestyle, diet, hygiene, infections, antibiotic use, sexual activity, and oestrogen have an impact on the microbiota composition. Recent research has highlighted a significant correlation between vaginal microbiota, immune status, and HPV infection [9]. In women of reproductive age, the normal vaginal microbiota is typically *Lactobacillus*-dominated, playing a protective role by maintaining low pH, producing antimicrobial compounds, and competitive exclusion [10]. Conversely, an increase in non-*Lactobacillus*-dominated microbiota such as *Sneathia*, *Atopobium*, or *Megaesphera* is associated with vaginal dysbiosis [11]. This dominance is reflected in the cervicovaginal environment, where microbial communities have been grouped into five main community state types (CSTs). Originally identified by Ravel et al. [10], CSTs I, II, III, and V are predominantly dominated by various *Lactobacillus* species. For instance, CST-I is characterised by the dominance of *L. crispatus*, CST-II by *L. gasseri*, CST-III by *L. iners*, and CST-V by *L. jensenii*. Alternatively, CST-IV is distinguished by a diverse microbiome composition, characterised by a higher abundance of non-*Lactobacillus* genera [10].

Abnormal vaginal environments are linked to the development and progression of cervical intraepithelial neoplasia (CIN) [12]. Although all CST types are observed across the different cervical stages including healthy, HPV-infected, LSIL (low-grade squamous intraepithelial lesion), or HSIL (high-grade squamous intraepithelial lesion), the distribution of these groups varies depending on the disease state. Significantly, as the disease stage progresses, the prevalence of CST-I and-V decreases, while CST-IV becomes more prevalent [13].

16S rRNA gene sequencing has become widely utilised to explore microbial diversity across various environments. These methods classify organisms based on phylogenetic differences reflected in their gene sequences, bypassing the need for cultivation [14]. The analysis of the 16S rRNA gene offers detailed insights into microbial populations such as species composition and abundance, and quantifies similarities and differences among microbial communities. The data obtained can be statistically analysed to assess changes over time or between groups. HPV testing using self-collected specimens (SCSs) offers comparable accuracy in detecting HR-HPV genotypes and women with a higher risk of CIN2 or worse [15,16]. However, little is known about the potential utility of self-collection on the determination of the microbiome composition using 16S rRNA gene sequencing methods, and on the identification of previously identified microbiome patterns associated with HPV infection.

Microbiome studies in cervicovaginal samples associated with HPV have mainly used liquid-based cytology (LBC) samples collected by healthcare professionals or alternatively, self-collection methods non-standardised for HPV detection [17].

In this study, we conducted a proof-of-concept analysis to evaluate the cervicovaginal microbiome and ascertain whether HPV-associated features exhibited comparable patterns between the samples collected by physicians with LBC and SCSs in a dry state.

## 2. Results

Fourteen out of the twenty-two paired SCS and LBC samples collected achieved sufficient sequencing depth for the study and met the sequencing quality criteria. Out of fourteen samples, eight were positive in the HPV test, while the remaining six were negative. The eight positive samples were positive for other HR-HPV types (neither 16 nor 18) (HPV-HR). Additionally, one of those samples was also positive for HPV16 (Supplementary Table S1). From the sequencing of the 16S rRNA gene, we obtained a total of 1,063,730 reads, with a median length per read of 1498 (IQR = 39) base pairs. The taxonomic assignment showed a total of 1036 genera, with a median of 131 (IQR = 88) genera per sample.

**Figure 1:**
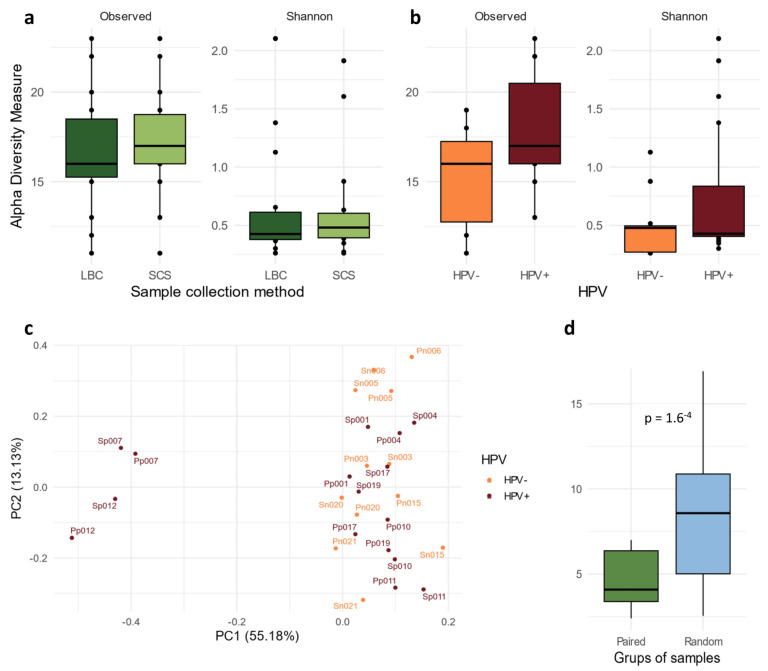
The α-diversity of the cervicovaginal microbiome comparing the species diversity detected in (**a**) LBC and SC samples and (**b**) in HPV-positive and -negative samples, as measured by both the observed and Shannon indices. (**c**) β-diversity analysis visualised in a PCA plot, showing the genera composition of each sample (paired samples are identified with the same ID). In orange, HPV− samples, and in red, HPV+. (**d**) Euclidian distance between samples obtained from the same patient was measured and compared with the distance between random pairs of samples. HPV: human papillomavirus; LBC: liquid-based cytology; SCS: self-collected samples; PC: principal component.

The α-diversity analysis showed no differences between the SCS and LBC samples (observed *p* = 0.41 and Shannon *p* = 0.73) (Figure 1a). HPV-positive samples showed a higher bacterial diversity than the HPV-negative samples (Figure 1b), although the differences did not reach significance (observed *p* = 0.10 and Shannon *p* = 0.39). We measured the similarity of the cervical microbiome composition (β-diversity) between both groups (LBC and SCSs). The PCA plot showed that paired samples (LBC and SCSs) exhibited close grouping (Figure 1c).

Euclidean distance was measured between both paired and random samples to quantify the degree of similarity in microbiome composition. The results revealed that the distances between paired samples were shorter in nearly 90% of the iterations (Figure 1d) than the distance obtained after 1000 random iterations (*p* < 0.001).

**Figure 2:**
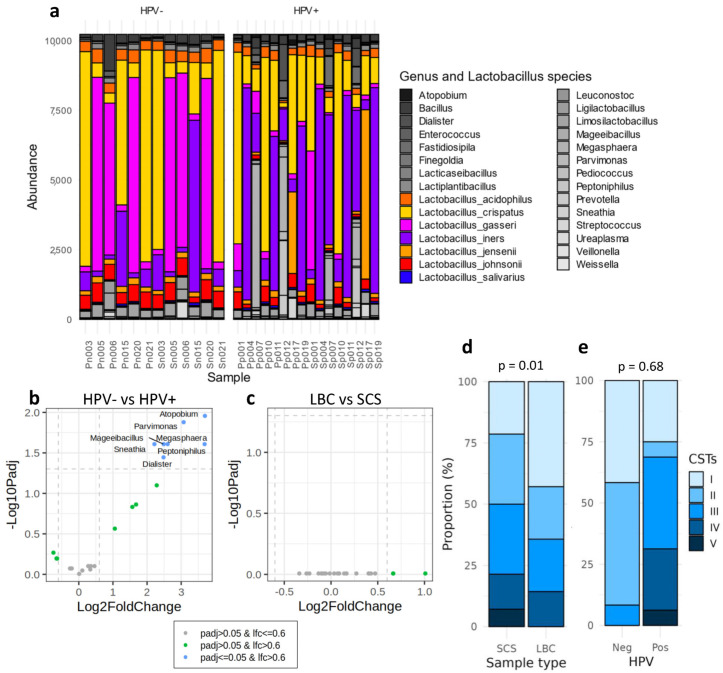
(**a**) Microbiome composition and abundance in HPV-negative and -positive samples. (**b**) Differential analysis at the genus level of the microbiome composition comparing HPV-positive vs. HPV-negative samples and (**c**) comparing the LBC vs. SC samples. Genera in blue show statistically significant differences in the HPV+ group (**c**) or in SCSs (**b**). In the (**b**,**c**) plots, dotted lines indicate the Log2(FC) limit (vertical) and the −Log10(p.adj) threshold (horizontal). In green, genera not statistically significant but with a log2(FC) higher than 0.6. (**d**,**e**) CST classification between (**d**) sample collection methods (SCS or LBC) and (**e**) HPV-positive or -negative samples. HPV: human papillomavirus; LBC: liquid-based cytology; SCS: self-collected samples; CSTs: community state types.

Analysis of the cervicovaginal microbiota revealed *Lactobacillus* as the predominant bacterial genus across all samples. As seen in the PCA plot, two distinct clusters of samples were identified (Figure 1c). The larger cluster comprised *Lactobacillus*-dominated samples, whereas the smaller cluster contained two samples where *Lactobacillus* was not dominant, and the other genus appeared with a higher abundance. However, the abundance of *Lactobacillus* was lower in the HPV-positive samples, which corresponded with an increase in the abundance of other genera (Figure 2a). Furthermore, a deeper analysis focusing on the *Lactobacillus* species composition identified distinct bacterial profiles between the two groups. A higher abundance of *L. crispatus* and *L. gasseri* were observed in the HPV-negative samples, whereas *L. inners* displayed the highest abundance in the HPV-positive samples (Figure 2a).

Beyond *Lactobacillus* abundance, differential microbiome analysis at the genus level between the HPV-positive and-negative samples (Figure 2b) revealed a statistically significant increase in the relative abundance of several genera in the HPV-positive samples. Specifically, *Atopobium* (*p.adj* = 0.011), *Parvimonas* (*p.adj* = 0.013), *Mageeibacillus* (*p.adj* = 0.025), *Sneathia* (*p.adj* = 0.025), *Megasphaera* (*p.adj* = 0.025), *Peptoniphilus* (*p.adj* = 0.025), and *Dialister* (*p.adj* = 0.036) exhibited notable increases (Supplementary Table S2). On the other hand, there were no discernible differences in bacterial abundance between the sample collection methods, implying that no genera were inflated or eliminated due to differences in the sampling process (Figure 2c).

No significant differences were found when comparing the CST classification between both sample collection methods (*p* = 0.68) (Figure 2d). In both the SC and LBC sample types, the community state types (CSTs) were equally assigned in 11 out of 14 samples. Discordant results were observed in three women, whose professional samples were classified as CST-I, and the SCSs as CST-II, CST-III, and CST-V. This discrepancy was caused by a shift in the dominant *Lactobacillus* species within the sample.

A difference in the CST groups were observed comparing the HPV-positive and -negative samples (*p* = 0.017) (Figure 2e). Most of the HPV-negative samples were categorized into CST-I or -II types. In contrast, all CST states were found in the HPV-positive samples, with CST-III the most prevalent. Notably, CST-IV and CST-V, commonly associated with vaginal alterations, appeared only in the positive samples (Figure 2e). From all of the samples analysed, only three HPV-positive women showed histological alterations. Two of them were diagnosed with LSIL and classified as CST-IV and CST-III, respectively, using both collection methods, while the sample with an HSIL was classified as CST-I by LBC and CST-V by SCS (Supplementary Table S1).

## 3. Discussion

This was a proof-of-concept study to determine whether there were differences in the cervicovaginal microbiome analysis when samples were collected by a healthcare professional or self-collected by the patient using the validated methods and devices for HPV-based cervical cancer screening [18]. Alpha- and beta-diversity analysis showed that the microbiome composition and abundance in the liquid-based cytology (LBC) samples collected by a professional were similar to that of the self-collected Evalyn Brush specimens (SCS).

A study by Forney et al. found that the composition of the vaginal microbiome remained similar regardless of the specific sampling device or location within the cervicovaginal subsite [17]. Despite the sampling methods possibly having an impact on the number of cells and DNA collected, the microbiome features were equivalent within the same patient. Other studies compared the microbial diversity between the self-collected swabs or tampons with the physician-collected samples [19,20]. Our results are aligned with previous research, indicating that no differences were found between the SCS and LBC samples, neither in the alpha nor beta diversity analyses (Figure 1b,c).

Moreover, the distinctive microbiome patterns associated with HPV infection or showing an HPV lesion remained discernible in both the SCSs and professionally collected LBC. In agreement with previous published studies [21,22], the HPV-positive samples were enriched in non-*Lactobacillus* genus such as *Atopobium*, *Parvimonas, Sneathia Megasphaera, Mageeibacillus, Peptoniphilus,* and *Dialister* (Figure 2b). Additionally, clear patterns were distinguished using the CST system in both the SCSs and professionally collected LBC. CST-I and CST-II were more prevalent in the HPV-negative women, while CST-III and CST-IV were dominant in the HPV-positive women (Figure 2d,e), as previously described [13]. Notably, one sample categorised as CST-IV, indicating vaginal dysbiosis, was identified as LSIL on cytology. To conclude, the microbiome composition is mainly influenced by individual or environmental factors, resulting in personalised variations within the cervicovaginal environment [20] instead of how the sample was collected.

Therefore, this study demonstrates that both validated HPV-testing methods commonly employed in cervical cancer screening programs can be equally utilised and implemented to assess the microbiome composition in women. Knowing this, we could consider testing the microbiome using leftover DNA from the collected samples for HPV testing. This opens the door for more research on how the microbiome might affect cancer progression. Moreover, this testing could be easily added to screening procedures, so there that there no need for extra samples or follow-up appointments after receiving an HPV-positive or abnormal cytology result.

We not only demonstrate the equivalence of both collection methods for microbiome detection, but we also identified microbiome HPV-associated features using leftover samples from screening, even after some time had passed since collection. Furthermore, the entire process, from DNA extraction to sequence including the bioinformatic analysis could be conducted in a period of 24-h in the same setting designed for HPV testing. Implementing a molecular test for the accurate triage of HPV-positive women using self-collected specimens could eliminate the need for referring them to primary care centres for a second sample collection for cytological analysis.

This study was a proof-of-concept that clearly shows that the SCS method could be good to analyse the microbiome after HPV detection in cervical cancer screening. The main limitation of the study was the low number of samples analysed; therefore, it would be of considerable interest to repeat it with an expanded sample size of patients to define in more detail the differences observed between SCSs and LBC as well as other aspects such as individual HPV genotyping. Moreover, we can optimise the protocol by increasing the running times and the number of reads per sample. Further research could validate the HPV-associated patterns observed in the microbiome and determine their sensitivity and specificity for identifying women with a HR-HPV infection who are at higher risk of developing CIN2 or more.

We assigned genus and an approximation of *Lactobacillus* species per sample, facilitating the classification of all samples into CSTs. This enabled the detection of more differences between the HPV-positive and -negative samples that could not be seen with only genera classification. However, 16 RNA seq methods for microbiome analysis are limited by errors in the assignation of species labels, and therefore, the CST classification [23]. We could explore the use of alternative sequencing tools to reduce the error rate, however, such methods could complicate the clinical implementation due to the need for specialised facilities. Conversely, self-sampling methods offer an ease of use and convenience, making them a promising option for further research. However, to gain a more detailed understanding of how a specific microbiome composition relates to cancer progression, larger studies with species-level resolution are needed to identify more specific markers.

The impact of the sample collection method on microbiome composition does not obscure the main features of the individual microbiome profile as our results suggest that SC and LBC samples are comparable in detecting distinctive microbiome profiles.

## 4. Methods and Materials

A total of 22 pairs of cervicovaginal samples (LBC and SCSs) were collected from women who participated in a study to evaluate self-sampling acceptability [18]. The LBC samples were collected by a healthcare professional using the Rovers Cervex-Brush and eluted in ThinPrep vials containing PreservCyt Solution (Hologic Inc., Marlborough, MA, USA), a medium designed for collecting and preserving cells, and DNA from the patient samples. SCSs were obtained by women using Evalyn Brush (Rovers Medical Devices B·V, Oss, The Netherlands), kept dry, and then eluted in 5 mL of PreservCyt Solution at the reference laboratory.

For the microbiome analysis, we used the leftover PreservCyt solution from the ThinPrep vials and the SC samples after HPV testing with the COBAS system (Roche Molecular Diagnostics, Indianapolis, IN, USA), which detects HPV16, HPV18, and other high-risk HPV (HR-HPV) genotypes. DNA from sample remnants of both sampling methods was isolated with the Maxwell^®^ 16 LEV Blood DNA Kit (Promega, Madison, WI, USA). The 16S rRNA gene sequencing libraries were prepared with the 16S Barcoding Kit (SQK-RAB204, Oxford Nanopore Technologies, Oxford, UK). The full-length 16S rRNA gene was amplified and barcoded from 30 ng input DNA previously extracted from the SCS and LBC samples, using LongAmp^®^ Taq 2× master mix (New England Biolabs, Ipswich, MA, USA) and pooled in equimolar amounts of amplicons per sample. Next, the library was incubated with Library Loading Beads, the mixture was added to Flow Cell version R.9.4, and sequenced using a MinIon sequencer for approximately 4–6 h (Oxford Nanopore Technologies—ONT, Oxford, UK).

The nanopore electrical signal was converted into nucleotides with MinKNOW GUI software (version 4.1.22) (Oxford Nanopore Technologies—ONT, Oxford, UK). Porechop (v0.2.4) [24] was applied to remove the adapters from the raw reads and to eliminate reads with adapters and barcodes in the middle of the sequence, while NanoFilt (v2.7.1) [25] was employed to delete reads falling outside the specified length range of 1300 to 1600 base pairs. Quality control was executed with FASTQC (v0.11.9) [26], and samples containing reads with an average quality score lower than nine were automatically excluded. Samples with less than 10,000 reads after the quality control were excluded. Taxonomy was assigned to the DNA sequences using Kraken2 (v2.1.2) [27] with a 16S rRNA gene reference database (SILVA 138 SSU Parc database) [28].

A customised filtering algorithm was applied to reduce the sequencing noise and taxonomical error. Genera with less than 100 counts in at least two samples out of the total number of samples were excluded. To adjust differences in the library size to aid diversity comparisons, a rarefaction was applied. Read counts were normalised by rarefying the dataset for all samples to a depth of 10,241 reads/sample with phyloseq (v1.42.0).

The α-diversity assessments were conducted to compare the genera richness between groups. To quantify the dissimilarities in the microbial community composition across samples and groups, we first normalised the dataset with the centred log-ratio transformation and then we applied β-diversity analysis with the Euclidian dissimilarity metric. The results were visualised using principal component analysis (PCA), which allowed for the exploration of patterns and relationships within the data. Differential abundance analysis of the microbiome compositional data was performed with the LINDA package, which fits linear regression models on the centred log-ratio transformed data.

For the CST classification, first, we selected all of the sequences previously classified as the *Lactobacillus* genus with kraken2. *Lactobacillus* species were classified using BLAST [29]. A local database was constructed with reference 16S rRNA sequences from the following *Lactobacillus* species: *L. acidophilus, L. crispatus, L. gasseri, L. iners, L. jensenii, L. johnsonii,* and *L. salivarius.* Reference sequences from *Lactiplantibacillus Plantarum, Limosilactobacillus reuteri, Limosilactobacillus fermentum,* and *Limosilactobacillus vaginallis* were used as controls. Subsequently, our set of samples underwent species classification using “blastn”, where homologous sequences were identified, and each read was assigned to a specific specie. Furthermore, utilising the 13,231 16S rRNA sequences compiled in the VALENCIA software (https://github.com/ravel-lab/VALENCIA/blob/master/CST_centroids_012920.csv, accessed on 22 April 2024) [30], we trained a random forest model for CST sample classification. Once CST prediction was accomplished, we proceeded to compare the CSTs between the HPV-positive and -negative samples as well as between the SC and LBC samples.

The significance level for all *p*-values was established at 0.05. Data analyses were carried out with R software version 4.2.1.

## Data Availability

The original contributions presented in the study are included in the article. Further inquiries can be directed to the corresponding authors.

## Supplementary Materials

The following supporting information can be downloaded at: https://www.mdpi.com/article/10.3390/ijms25115736/s1.
