# Supplementary material for: Proof of Concept Study: Comparability of Microbiome Diversity in Self- and Physician-Collected HPV-Positive and HPV-Negative Cervicovaginal Samples
Source: Int J Mol Sci. 2024 May 24;25(11):5736. doi: 10.3390/ijms25115736 (PMC11172023; doi:10.3390/ijms25115736)
Supplement: Supplementary file 1 [file ijms-25-05736-s001.zip › ijms-3002852-supplementary.pdf]

SUPPLEMENTARY DATA

Proof of Concept Study: Comparability of Microbiome Diversity in Self- and Physician-Collected HPV-Positive and HPV-Negative Cervicovaginal Samples

Laura Asensio-Puig, Álvaro de Andres-Pablo, Olfat Khannous-Lleiffe, Raquel Ibañez, Amelia Acera, Silvia de Sanjosé Toni Gabaldón, Laia Alemany, Laia Bruni, Miquel Àngel Pavón

Table S1: Patient characteristics alongside their HPV status (positive/negative), genotype, cytology results, and CST classification by LBC and SCS.

Patients	HPV	HPV-test	Cytology	CSTs	
				LBC	SCS
1	Positive	HR-HPV	Negative	I	II
3	Negative	-	Negative	I	I
4	Positive	HR-HPV	Negative	III	III
5	Negative	-	Negative	II	II
6	Negative	-	Negative	II	II
7	Positive	HR-HPV	Negative	IV	IV
10	Positive	HR-HPV	Negative	I	I
11	Positive	HR-HPV	Negative	III	III
12	Positive	HPV16, HR-HPV	LSIL	IV	IV
15	Negative	-	Negative	I	III
17	Positive	HR-HPV	HSIL	I	V
19	Positive	HR-HPV	LSIL	III	III
20	Negative	-	Negative	II	II
21	Negative	-	Negative	I	I

HR-HPV: high-risk human papillomavirus; LSIL: low-grade squamous intraepithelial lesion; HSIL: high-grade squamous intraepithelial lesion; LBC: liquid-based cytology; SCS: self-collected samples; CSTs: community state types.

Table S2: Differential expression analysis comparing the expression levels of various genera between the HPV+ and HPV- samples. Values with a log2FC greater than 0.6 and with a p-adjusted by FDR (False Discovery Rate) lower than 0.05 are highlighted in grey.

Genera	Log2FC	P.val	P.adj
Atopobium	3.695	0.001	0.011
Megasphaera	3.686	0.006	0.025
Parvimonas	3.072	0.001	0.013
Peptoniphilus	2.603	0.006	0.025
Mageeibacillus	2.486	0.004	0.025
Dialister	2.482	0.011	0.036
Fastidiosipila	2.283	0.028	0.079
Sneathia	2.215	0.006	0.025
Veillonella	1.678	0.054	0.137
Prevotella	1.569	0.064	0.147
Finegoldia	1.056	0.130	0.272
Streptococcus	0.458	0.535	0.791
Pediococcus	0.348	0.584	0.791
Ureaplasma	0.336	0.794	0.870
Bacillus	0.275	0.559	0.791
Enterococcus	0.104	0.857	0.896
Lactiplantibacillus	0.010	0.987	0.987
Weissella	-0.206	0.735	0.845
Lacticaseibacillus	-0.239	0.704	0.845
Lactobacillus	-0.258	0.726	0.845
Leuconostoc	-0.637	0.388	0.638
Limosilactobacillus	-0.653	0.381	0.638
Ligilactobacillus	-0.744	0.282	0.541
